# Voluntary HIV counselling and testing among men in rural western Uganda: Implications for HIV prevention

**DOI:** 10.1186/1471-2458-8-263

**Published:** 2008-07-30

**Authors:** Francis M Bwambale, Sarah N Ssali, Simon Byaruhanga, Joan N Kalyango, Charles AS Karamagi

**Affiliations:** 1 Clinical Epidemiology Unit, Makerere University, P.O.Box 7072, Kampala, Uganda; 2 Department of Women and Gender Studies, Makerere University, P.O.Box 7062, Kampala, Uganda; 3 Hoima Regional Referral Hospital, P.O.Box 5, Hoima, Uganda; 4 Department of Pharmacy, Makerere University, P.O.Box 7072, Kampala, Uganda; 5 Department of Paediatrics and Child Health, Makerere University, P.O.Box 7072, Kampala, Uganda

## Abstract

**Background:**

Voluntary HIV counselling and testing (VCT) is one of the key strategies in the prevention and control of HIV/AIDS in Uganda. However, the utilization of VCT services particularly among men is low in Kasese district. We therefore conducted a study to determine the prevalence and factors associated with VCT use among men in Bukonzo West health sub-district, Kasese district.

**Methods:**

A population-based cross-sectional study employing both quantitative and qualitative techniques of data collection was conducted between January and April 2005. Using cluster sampling, 780 men aged 18 years and above, residing in Bukonzo West health sub-district, were sampled from 38 randomly selected clusters. Data was collected on VCT use and independent variables. Focus group discussions (4) and key informant interviews (10) were also conducted. Binary logistic regression was performed to determine the predictors of VCT use among men.

**Results:**

Overall VCT use among men was 23.3% (95% CI 17.2–29.4). Forty six percent (95% CI 40.8–51.2) had pre-test counselling and 25.9% (95%CI 19.9–31.9) had HIV testing. Of those who tested, 96% returned for post-test counselling and received HIV results. VCT use was higher among men aged 35 years and below (OR = 2.69, 95%CI 1.77–4.07), the non-subsistence farmers (OR = 2.37, 95%CI 2.37), the couple testing (OR = 2.37, 95%CI 1.02–8.83) and men with intention to disclose HIV test results to sexual partners (OR = 1.64, 95%CI 1.04–2.60). The major barriers to VCT use among men were poor utilization of VCT services due to poor access, stigma and confidentiality of services.

**Conclusion:**

VCT use among men in Bukonzo West, Kasese district was low. In order to increase VCT use among men, the VCT programme needs to address HIV stigma and improve access and confidentiality of VCT services. Among the more promising interventions are the use of routine counselling and testing for HIV of patients seeking health care in health units, home based VCT programmes, and mainstreaming of HIV counselling and testing services in community development programmes.

## Background

In Uganda, HIV/AIDS remains a major public health problem, mainly affecting people in the productive and reproductive age group of 15 to 49 years. About 1.2 million people are living with HIV while 1.8 million people have died of HIV/AIDS [1]. However, Uganda has reversed the HIV sero-prevalence from 30% in 1990 to 6.5% in 2003 [2]. This remarkable success has been achieved through promotion of the *ABC *strategy (*abstinence, be faithful, condom use*), effective treatment of opportunistic infections, prevention of mother-to-child transmission of HIV (PMTCT), use of antiretroviral therapy (ART) and HIV voluntary counselling and testing (VCT) [2]. During 2005, the national ART programme was operational in hospitals but had not been rolled out to the health sub-districts.

HIV voluntary counselling and testing (VCT) is now widely accepted as the cornerstone of HIV prevention programmes in many countries because of its multiple benefits [3-8]. Furthermore, VCT is the gateway to comprehensive HIV care and support including access to antiretroviral therapy [9]. Newer approaches of VCT delivery including routine HIV counselling and testing[10], home-based VCT[11,12], use of community-based lay counsellors[13], couple counselling and testing[14], and same-day mobile VCT[15,16], have been added to the traditional VCT delivery systems of free standing, health unit based, and outreach VCT services [17].

Despite the array of delivery approaches and the advantages of VCT, uptake in sub-Saharan Africa is disappointingly low with reports of 12% to 56% among couples or the general public [9,18-22]. Furthermore, there is very little information on VCT uptake among men, and also on the factors that influence it [2,23]. It is not known whether factors that influence VCT uptake in the general population are also operational in VCT uptake by men only [10-15,21,24,25]. Men's utilization of VCT is important because in many societies including those in Uganda, men are the heads of households and control decisions and resources that are essential for HIV prevention and care. As Uganda gears to consolidate gains in HIV prevention, it is vitally important that men are fully involved in the HIV prevention and control strategies. It is against this background that we conducted a study to determine the prevalence and factors associated with VCT utilization among men in Kasese district, Uganda.

## Methods

### Study site

Kasese District is situated in Uganda's western region and is divided into two counties namely Bukonzo and Busongora. It had a population of 530,000 people in 2005, of which 80% were rural. The population is predominantly *Bakonzo *and the main language is *Lhukonzo*. The literacy rate was 37% for men and 33% for women, and the main economic activity was subsistence farming [26]. In 2005, the HIV prevalence was reported to be 13.3% [2]. The study site was Bukonzo West health sub-district (HSD). Bukonzo West HSD has two static VCT sites namely Bwera hospital and Kasanga health centre III that provide preventive and curative outpatient services. It also has a few outreach VCT sites in some of the parishes. The HSD consists of 5 sub-counties, 32 parishes and 170 villages and is predominantly a rural setting. It has a total population of about 123,967 persons of which 48% are males. The mean household size is 5.1, and on average, 3 in 4 households are headed by males [27].

### Study design, population and sampling

**Figure 1:**
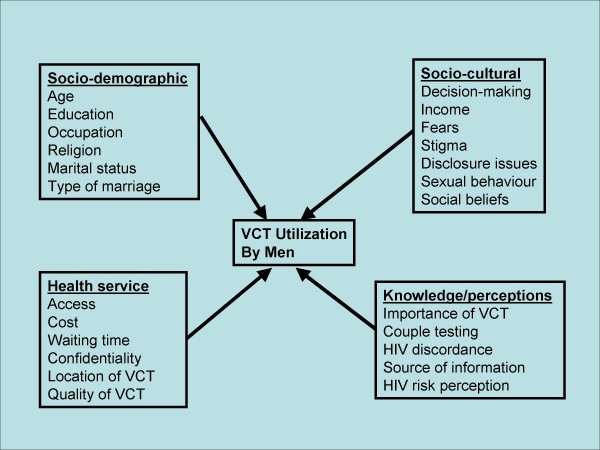
Conceptual framework of VCT utilization by men and possible predictor variables among 780 men in Kasese district, Uganda, 2005.

The study was a population-based cross-sectional study employing quantitative and qualitative methods of data collection and conducted between January and April 2005. Triangulation of the quantitative and qualitative research methods and data were used for validation and completeness of data[28]. We used a conceptual framework consisting of independent predictors of VCT including socio-demographic, socio-cultural, and health service factors as well as knowledge and perceptions on VCT to design the instruments and to guide the analysis (Figure 1).

### Quantitative study

The study participants were adult men aged 18 years and above, residing in Bukonzo West HSD during the study period, and who consented to participate in the study. We excluded men who were very sick, deaf or mentally ill. Cluster sampling and simple random sampling were used to obtain the sample. A cluster was defined as a village. A list of villages (Local Council I) in Bukonzo West health sub-district was obtained from the District Planning unit. A total of 170 villages were numbered according to the order in which they appeared on the list to form a sampling frame.

For each village, the cumulative population was calculated. A sampling interval was calculated by dividing the total population by the number of clusters required in the sample [38]. A random number not exceeding the sampling interval was then chosen using a table of random numbers. The first cluster selected was the village in whose cumulative population the random number fitted. Subsequent clusters were selected by adding the sampling interval to the previous population. Thus a sample of 38 clusters (villages) was selected by using probability proportional to size (PPS).

In each village, the households were randomly selected using a list of heads of households provided by Local Council chairpersons of the respective villages. The principal investigator and research assistants, with the help of a guide walked around the selected villages. Eligible household members who consented to participate in the study were interviewed. A maximum of 25 households were randomly selected for each village. If in a household there was more than one eligible person, one of them was randomly selected for the interview. Households where eligible men could not be found were skipped.

### Sample size estimation

The sample size was estimated using the modified Kish and Leslie (1965) formula. The prevalence of VCT use among men was estimated at 50% to give the highest power possible and hence the lowest sample size. A design effect of two was incorporated in the formula to cater for the design effect due to the cluster sampling method. The number of clusters C, was calculated using the formula C = [p (1 - P) D]/S^2^b, where b = number of responses per cluster (village) = 21 (on average), and S = standard error = √[P (1-P) D/n] [29]. The required number of clusters was 38 and the sample size was estimated at 780 participants.

### Variables

The independent variables included socio-demographic characteristics (age, marital status, occupation, education level, type of marriage, religion and income), knowledge and perceptions (knowledge of VCT, source of information about VCT, perceived risk of HIV, perceived benefit of VCT, discordance and importance of couple testing), socio-cultural factors (decision-making, sexual behaviour, stigma, fear of rejection by sexual partner, couple testing, disclosure of test results and social beliefs), and health service factors (willingness to pay, distance to VCT site, travel times, availability of VCT service, confidentiality, and quality of service). The outcome variable for the study was VCT use and was measured at three levels: pre-test counseling, HIV testing, and post-test counseling and receiving HIV test results. VCT use was considered complete if the participant received all three.

The principal investigator and research assistants administered a pre-tested semi-structured questionnaire to eligible men. The instruments were translated into *Lhukonzo *and back translated into English. Twelve research assistants were trained and commissioned. These were mainly men who had attained Senior Four education (11 years), were fluent in *Lhukonzo *and English, and were residents of Bukonzo West HSD. Throughout data collection, regular meetings were held with the research assistants in order to edit the completed questionnaires and to review field experiences and performance so as to improve subsequent interviews. Data collected were kept under lock and key by the principal investigator.

### Data analysis

Quantitative data was entered into EPINFO version 6.04 and then exported to Stata version 8.0 for analysis that adjusted for the design effect. Unadjusted analysis was performed between VCT use by the men as the outcome variable and each independent variable. Variables that were significant (p-value < 0.05) were then entered into a model for logistic regression using the backward elimination method so as to control for confounding. The criteria used for the backward elimination model was the default of a p-value > 0.10. Associations between the outcome and independent variables were assessed using odds ratios, 95% confidence limits and p-values. A p-value of 0.05 or less was taken to be statistically significant.

### Qualitative study

Four focus group discussions consisting of 10–12 participants per group were conducted in *Lhukonzo *by the Principal Investigator as the moderator assisted by a note taker. We used a focus group discussion guide composed of predetermined topics including awareness of VCT services provided in the area; importance of VCT; reasons that influence people to seek VCT; VCT utilization by men; and socio-cultural beliefs that influence VCT utilization among men. The FGDs were chosen strategically to represent variations in the age and place of residence. The FGD participants were stratified by sex and included men, women, and VCT clients who were aged 18 years and above. Two FGDs for men, one FGD for women and one FGD for VCT clients at Bwera hospital were conducted. The women were included in this study in order to capture their opinions and views about the men since men have an influence on them in regard to decisions about VCT use. The FGDs were meant to provide a deeper understanding of the information on the perceptions and socio-cultural aspects of the study. The information shared was recorded on audio tapes and notes taken. The data collected was transcribed verbatim and translated from *Lhukonzo *into English and analyzed manually according to themes arising. The data was then triangulated with the quantitative findings from the individual household interviews.

Ten key informant interviews were held with two Community Health Workers, one religious leader, two NGO managers, one Community Based Organization manager, two VCT counsellors, the District Director of Health Services and the in-charge of Bukonzo West HSD. The interviews were carried out using an open-ended questionnaire that was presented to the informant shortly before the interview. After transcription and translation, the data was coded manually. Data from the focus group discussions and key informant interviews was then triangulated with the quantitative findings.

### Ethics

Makerere University Clinical Epidemiology Unit, the Faculty of Medicine Ethics and Research Committee and the Uganda National Council of Science and Technology approved the study. The research participants were enrolled in the study after written informed consent. The participants were informed that their participation in the study was voluntary and they could withdraw from the study at anytime. In addition, measures were taken to ensure the confidentiality of the data. In particular, the interviews were held in privacy, away from relatives and friends; information on HIV status was not collected; codes rather than names of participants were used; and the data was kept under lock and key by the principal investigator.

## Results

### Characteristics of the respondents

Between January and April 2005, a total of 780 men were enrolled into the study (Table 1). The men were aged between 18 and 90 years with a median age of 32 years. Of the 780 men, 45.3% were Roman Catholic, 43.7% were Anglican while 6.7% were Muslim. Most of the men (71.3%) were married. Of these, 76.3% were in monogamous unions. The majority of the men were peasant farmers (58.1%), and 32% were earning less than one dollar a day (1US$ ≈ 1700 Uganda Shillings).

**Table 1:** Frequency of voluntary counselling and testing among 780 men in Kasese district, Uganda, 2005

**Variable**	**Total** **n (%)**	**Accepted ** **VCT**	**Percent** **(95% CI)**
**Overall**	**780**	**182**	**23 (17–29)**
**Age**			
≤ 20	86(11)	13	15
21–29	237(30)	81	34
30–39	242(31)	62	26
40–49	121(16)	23	19
50+	94(12)	3	3
**Education**			
None	178(23)	22	18
Primary	309(40)	56	18
Secondary	196(25)	53	27
Tertiary	97(12)	41	42
**Religion**			
Roman Catholic	353(45)	75	21
Anglican	341(44)	84	25
Muslim	52(07)	14	27
Other	97(12)	9	27
**Occupation**			
Subsistence farmer	453(58)	76	17
Salaried employee	119(15)	47	40
Businessman	105(14)	31	30
Student	94(12)	26	28
Other	9(01)	22	2
**Marital status**			
Single	209(27)	59	28
Married	556(71)	122	22
Separated/divorced	15(02)	1	7
**Type of marriage**			
Monogamy	432(55)	101	23
Polygamy	134(17)	21	16

### Prevalence of VCT use among men

Of the 780 men interviewed, 46 % (95% CI 40.8–51.2) had pre-test counselling while 25.9 % (95% CI 19.9–32) had tested for HIV. Thus the VCT drop out was 43.7 %. Of the 201 men who tested for HIV, 96% received their HIV test results. The prevalence of complete VCT use (pre-test counselling, HIV testing, post test counselling and receiving HIV test results) was 23.3% (95% CI 17.2–29.4) (Table 1). The most common reason for taking an HIV test was to know the HIV sero-status (74.3%) followed by frequent illness (15.3%) and plans for marriage (15.3%). When asked if they were willing to seek VCT in the near future, the majority (79%) said they were willing to go for VCT. However, willingness to seek VCT in the future varied among the two groups being 74.9% among those who had never had VCT at all and 83.9% among those who had ever had VCT.

### Knowledge and perceptions about VCT

The majority (80.1%) of the men said they were aware about the existence of the VCT program in their area. When asked what was involved in the VCT process, they mentioned pre-test counselling on HIV (78.6%), taking off blood for HIV testing (92.6%) and counselling on the prevention and control of HIV/AIDS (81.6%). Most men (98.7%) knew of a site offering VCT services in Bukonzo West. The majority of the men received information about VCT through the health workers (75.2%) but other sources of information included friends (12.8%), radio (13.9%), newspapers (6.9%), and community leaders (5.4%). Of the 780 participants interviewed, 94.9% said that VCT was important for the good of the individual and the family. However, most men were worried of taking an HIV test because to them having a positive result meant imminent death.

*"You can die very soon if you tested positive because of worries" (Male, Nyakiyumbu FGD)*.

Only 9.1% of the men perceived themselves to be at a high risk of HIV and yet in the FGDs, the majority of the men, especially single men, considered themselves to be at high risk of HIV because of multiple sexual relationships.

*"I have tested several times and been found negative. But this time am worried of my status because of the fourth wife whom I married recently" (Male 33 years, Karambi FGD)*.

Of the 780 respondents interviewed, 91.3% said that it was important to test together with their sexual partners. The findings from the FGDs showed that most men appreciated the importance of couple testing, with the majority saying that it would help them in family planning in the event of a positive HIV test result. On the contrary, men said that going to the health facility for HIV testing with a spouse was not common in their culture and would be considered as strange behaviour.

On the issue of discordance, over half of the men (61.8%) did not believe that discordant HIV results could exist. This view was supported by the FGDs where most men said it was not possible for one of the cohabiting partners to test HIV positive and the other HIV negative. They believed that if one of the partners was found HIV positive, then it was automatic that the other also had HIV infection.

*"Some men have lost their wives due to HIV/AIDS but they lie to us that they were found HIV negative when they tested. This is not true" (Male, 35 years old, Nyakiyumbu FGD)*.

### Socio-cultural factors

On disclosure of HIV test results, the majority of the men (81.8%) said they would disclose to their partners. There was, however, contradiction in the FGDs whereby the majority of the men felt that disclosure of HIV positive results would deny them their sexual rights and would imply that they brought the disease into the family.

"Some of our women are very emotional and if you disclosed to her, she might end up denying you your sexual rights" (Male, 34 years old, Key informant)

With regard to sexual partners, 35.1% of the men reported having two or more sexual partners. During the past one year, 24.9% of the men reported having had extramarital sex. Of those who had extra-marital sex, 76.8% used a condom on their last sexual encounter. Both men and women in the focus group discussions confirmed that multiple sexual relationships were common among the men and this could negatively affect VCT use. The participants felt that having VCT would not stop men from acquiring more sexual partners. Most participants said that the majority of the men live in doubt that they might be infected because of having multiple sexual partners.

*"Men have many sexual partners and fear that they might be infected with HIV already" (Female, Kyasenda FGD)*.

Participants were asked if they had any fears about taking an HIV test and 41.3% said they had fear of taking the HIV test. When asked to mention the fears they had, they mentioned inaccuracy of the HIV testing (37.9%), stigma (57.1%), fear of divorce or separation from partner (38.8%) and lack of confidentiality (8.4%). The participants further mentioned that the majority of the men felt that if they tested positive, their families and the society would easily tell that they were infected. They expressed the feeling that the community might look at them differently and also deny them certain rights like holding political positions. Similar findings were obtained during the focus group discussions.

The participants were asked if they would feel comfortable if they were seen at the VCT site and the majority (83.1%) said they would feel comfortable. The commonest reasons given for feeling uncomfortable were fear of being labelled an HIV victim (67.4%), stigma (36.4%), and meeting a relative or familiar people (11.4%). These findings were also supported by the focus group discussions.

The participants were asked if they had any socio-cultural beliefs that might influence men to seek VCT services. Only 20% said that there were social beliefs that would influence men to seek VCT. Of those who said beliefs existed, the majority mentioned men's superiority over women (30.8%), if partner (wife) tested positive, then the man was also automatically positive (18.8%), widow inheritance/many sexual partners (17.3%) and witchcraft (14.7%). During the FGDs, the commonest belief mentioned was that of the superiority of men. Men superiority had a negative influence on the VCT seeking behaviour. Most decisions regarding health seeking behaviour among family members are dominated by the men and in most cases what the man decides is unchallenged by the entire family. Men also felt that once their sexual partners tested for HIV, they did not need to seek VCT because their results would be the same as those of their partners

Participants were asked whom they would consult before deciding to go for VCT. Of the 780 respondents, 40.9% said they would consult their partners/spouses, followed by friends (15.8%), health workers (15.0%) and 22.7% said they would not consult any body. The participants were asked what action they would take if they tested HIV positive. The majority said they would abstain from sex (33.8%), live positively (29%), seek treatment/join The AIDS Support Organisation (TASO) (24.5%), or simply keep quiet (5.9%).

### Health service factors

Most men expressed the feeling that they would go somewhere else other than in the HSD, the majority preferring VCT sites outside Bukonzo West where the VCT counsellors do not know them. They feared that they would easily be identified and labelled as HIV victims. Some men also said that the VCT staff were being bribed and hence giving negative results to HIV positive clients.

"I don't trust those health workers because they might not keep people's secrets" (Male, Bwera FGD)

" I would prefer that the VCT Counsellors be people from other areas other than from Bukonzo" (Male & Single, FGD Karambi)

A sizeable proportion of the respondents (44.7%) said they would be unwilling to pay for the VCT services.

Of the 201 men who had ever undergone VCT, the main reasons given for choosing a particular VCT site were confidentiality (56.2%), proximity (37.8%), convenience (1.0%) and other (5.0%). The majority of the participants (91.6%) who had ever undergone VCT said they were satisfied with the VCT services. The majority of the participants (69%) lived within a radius of 5 kilometres from the nearest static VCT site. The commonest means of transport to the VCT sites was by walking (69.6%).

During the FGDs, the majority of the participants said that the VCT sites were few (only two) and were located very far from the people, hence making the VCT services inaccessible. Most of the men wanted VCT services to be extended to the villages or to their homes in order to save them the costs of time and transport.

### Association between independent factors and VCT use among men

On unadjusted analysis, age 35 years or less (OR = 2.97, 95% CI 2.00–4.43); secondary or tertiary education (OR = 2.10, 95% CI 1.50–3.00); non-subsistence occupation (OR = 2.54, 95% CI 1.68–3.85); willingness to test for HIV with partner (OR = 5.33, 95% CI 1.92–14.85); willingness to disclose HIV results to partner (OR = 2.19, 95% CI 1.43–3.38); feeling comfortable with VCT site (OR = 2.71, 95% CI 1.54–4.77); wanting to know HIV status (OR = 0.19, 95% CI 0.13–0.27); and a monthly income of more than 50,000 Uganda Shillings (OR = 1.84, 95% CI 1.23–2.76) were associated with VCT use among men (Table 2).

**Table 2:** Unadjusted association between independent factors and voluntary counselling and testing among 780 men in Kasese district, Uganda, 2005

**Variable**	**No VCT ** **n (%)**	**Accepted VCT ** **n (%)**	**OR (95% CI)**
**Age in years**			
>35	253(88)	36(13)	1.0
≤ 35	345(70)	146(30)	2.97(2.00–4.43)
**Education**			
None/Primary	399(82)	88(18)	1.0
Secondary/Tertiary	199(68)	94(32)	2.10(1.50–3.00)
**Religion**			
Muslim	38(73)	4(27)	1.0
Christian	560(77)	168(23)	0.81(0.43–1.54)
**Occupation**			
Subsistence farmer	526(80)	135(20)	1.0
Other	72(77)	47(23)	2.54(1.68–3.85)
**Marital status**			
Not married	164(73)	60(27)	1.0
Married	434(78)	122(22)	0.77(0.54–1.10)
**Type of marriage**			
Monogamy	331(77)	101(23)	1.0
Polygamy	133(84)	21(16)	0.61(0.36–1.02)
**Fear of taking an HIV test**			
No	327(71)	131(29)	1.0
Yes	271(84)	51(16)	0.47(0.33–0.67)
**Fear of divorce or separation**			
No	144(82)	32(18)	1.0
Yes	127(87)	19(13)	0.67(0.36–1.25)
**Lack of confidentiality**			
No	245(83)	50(17)	1.0
Yes	26(96)	1(04)	0.19(0.03–1.42)
**Willingness to test for HIV with partner**			
No	64(94)	4(06)	1.0
Yes	534(75)	178(25)	5.33(1.92–14.85)
**Willingness to disclose HIV results to partner**			
No	181(86)	30(14)	1.0
Yes	417(73)	152(27)	2.19(1.43–3.38)
**Would feel comfortable at VCT site**			
No	117(88)	15(11)	1.0
Yes	481(74)	167(26)	2.71(1.54–4.77)
**Wanting to know HIV sero-status**			
No	115(54)	98(46)	1.0
Yes	348(86)	55(14)	0.19(0.13–0.27)
**Monthly income (Uganda Shillings)**			
≤ 50,000	482(79)	131(21)	1.0
>50,000	90(67)	45(33)	1.84(1.23–2.76)
**Number of sexual partners**			
One	382(76)	141(24)	1.0
Two or more	216(79)	58(21)	0.83(0.58–1.18)
**Had sex outside marriage in past 1 year**			
No	457(78)	129(22)	1.0
Yes	141(73)	53(27)	1.33(0.92–1.93)
**Used a condom on last sexual encounter**			
No	32(71)	13(29)	1.0
Yes	109(73)	40(27)	0.90(0.43–1.20)
**Social belief exists**			
No	481(77)	143(23)	1.0
Yes	117(75)	39(25)	1.12(0.75–1.68)
**Willingness to seek VCT**			
No	135(82)	29(18)	1.0
Yes	463(76)	153(25)	1.54(0.99–2.39)
**Distance to nearest VCT site 5 km or less**			
No	187(77)	55(23)	1.0
Yes	411(76)	127(24)	1.05(0.73–1.50)

On logistic regression with complete VCT as the outcome, age (OR = 2.89, 95% CI 1.77–4.07), occupation (OR = 2.37, 95% CI 1.52–3.71), fear of taking an HIV test (OR = 0.54, 95% CI 0.37–0.79), testing together with a spouse/partner (OR = 3.01, 95% CI 1.02–8.83), and willingness to disclose HIV test results to their sexual partners (OR = 1.64, 95% CI 1.04–2.60) were independently associated with VCT use among men (Table 3). Table 4 shows results of logistic regression of factors associated with seeking pre-test counselling that included: to know the HIV sero-status (OR = 12.25, 95% CI 6.09–24.64); feeling comfortable with the VCT site (OR = 1.75, 95% CI 1.09–2.83); fear of being stigmatized (OR = 1.84, 95% CI 1.10–3.08); non-subsistence farmer (OR = 1.90, 95% CI 1.35–2.67); and confidentiality (OR = 15.91, 95% CI 5.54–45.74). Table 5 shows results of logistic regression of factors associated with complete VCT among men who went for pre-test counselling that included: more than 7 years of education (OR = 2.9, 95% CI 1.1–7.8); to know the HIV sero-status (OR = 84.6, 95% CI 23.3 – 306.4); fear of inaccurate HIV test result (OR = 30.7, 95% CI 2.1 – 457.4); and confidentiality (OR = 53.1, 95% CI 10.8–261.8).

**Table 3:** Logistic regression of independent factors associated with acceptance of VCT among 780 men in Kasese district, Uganda, 2005

**Variable**	**No VCT ** **n (%)**	**Accepted VCT ** **n (%)**	**OR ** **(95% CI)**
**Age in years**			
>35	253(88)	36(13)	1.0
≤ 35	345(70)	146(30)	2.69(1.77–4.07)
**Occupation**			
Subsistence farmer	526(80)	135(20)	1.0
Other	72(77)	47(23)	2.37(1.52–3.71)
**Fear of taking an HIV test**			
No	327(71)	131(29)	1.0
Yes	271(84)	51(16)	0.54(0.37–0.79)
**Willingness to test for HIV with partner**			
No	64(94)	4(06)	1.0
Yes	534(75)	178(25)	3.01(1.02–8.83)
**Willingness to disclose HIV results to partner**			
No	181(86)	30(14)	1.0
Yes	417(73)	152(27)	1.64(1.04–2.6)

**Table 4:** Logistic regression of independent factors associated with pre-test counselling among 780 men in Kasese district, Uganda, 2005

**Variable**	**Pre-test counselled ** **n (%)**	**Not pre-test counselled ** **n (%)**	**OR (95% CI)**
**To know HIV sero-status**			
No	411(65.2)	219(34.8)	1.0
Yes	10(6.7)	140(93.3)	12.25 (6.09–24.64)
**Feeling comfortable with VCT site**			
No	91(68.9)	41(31.1)	1.0
Yes	330(50.9)	318(49.1)	1.75 (1.09–2.83)
**Fear of being stigmatized**			
No	312(52.3)	284(47.7)	1.0
Yes	109(59.2	75(40.8)	1.84 (1.10–3.08)
**Occupation**			
Subsistence farmer	279(61.6)	178(38.4)	1.0
Other	142 (43.4)	185(56.6)	1.90 (1.35–2.67)
**Reason for choosing VCT site**			
Other	417(62.5)	250(37.5)	1.0
Confidentiality	4(3.5)	109(96.5)	15.91 (5.54–45.74)

**Table 5:** Logistic regression of independent factors associated with complete VCT among 369 men seeking VCT in Kasese district, Uganda, 2005

**Variable**	**Pre-test counselling only ** **n (%)**	**Complete VCT ** **n (%)**	**OR (95% CI)**
**Education in years**			
0 – 7	131(59.5)	89(40.5)	1.0
More than 7	55(36.9)	94(63.1)	2.9 (1.1–7.8)
**To know HIV sero-status**			
No	181(79)	48(21)	1.0
Yes	5(3.6)	135(96.4)	84.6 (23.3 – 306.4)
**Fear of inaccurate HIV test result**			
No	152(46.9)	172(53.1)	1.0
Yes	34(75.6)	11(24.4)	30.7 (2.1 – 457.4)
**Reason for choosing VCT site**			
Other	184(70.8)	76(29.2)	1.0
Confidentiality	2(1.8)	107(98.2)	53.1 (10.8–261.8)

## Discussion

### Prevalence of VCT use among men

The prevalence of VCT use among men in Bukonzo West was low at only 23.3%. Our findings are similar to those of other population-based studies in sub-Saharan Africa. De Graft and colleagues examined use of VCT services in a rural district population of Malawi and found that 11% of men had ever been tested for HIV [18]. Similarly, in an analysis of data from a population-based survey and a government clinic survey in the Eastern Cape Province of South Africa on VCT services, Hutchinson and Mahlalela reported that 17% of men had been tested for HIV [20]. Our findings confirm that VCT uptake among men is low and reinforce the widely reported observation that men are not fully involved in HIV prevention programmes.

### Factors associated with VCT use among men

#### Socio-demographic characteristics

Consistent with the study by Hutchinson and colleagues in Eastern Cape, South Africa [20], older men (35 years or more) were less likely to use VCT than younger men. In our study population, older men tended to be more conservative and may not have accepted VCT readily. They were more likely to be rural subsistence farmers, less educated, and less informed about HIV and VCT. Furthermore, they were more likely to be married, with less risky sexual behaviour, and thus with a lower perception of HIV risk. Because of their lower education and stronger roots to tradition, they were more likely to hold fatalistic attitudes about HIV and to prefer to remain in a state of denial regarding their HIV status. In Ghana, individuals with no formal education were more likely to stigmatize HIV/AIDS [13].

### Knowledge and perceptions on VCT

Although most men were familiar with the VCT programme and its procedures, this knowledge did not translate into high VCT use. Our findings contrast with those of Sherr and colleagues who reported that in a rural Zimbabwe cohort, motivation for VCT was driven by knowledge on VCT [25]. The discrepancy between knowledge and practice in our study may be due to the frequently reported knowledge-practice gap. However, it could also be an indicator of critical knowledge gaps on HIV including VCT among the men. In support of this, most men in our study did not know about HIV discordance, although understanding of discordance even among discordant people is extremely low [3,14]. Men's gaps in HIV knowledge combined with superstition may explain why fatalism was a barrier against VCT use among men.

AIDS related stigmas created barriers to seeking VCT among the men. More than half of the men feared to test for HIV because of stigma. Men were worried of being labelled HIV-infected because they would lose their social privileges. They expressed fear of meeting familiar people in HIV testing clinics, and preferred to test in distant clinics where they were not known by the people and staff. Our findings are consistent with research in sub-Saharan Africa which shows AIDS related stigmas are important barriers to VCT utilization [12,15,20,21,25]. As demonstrated by Wolff and colleagues in Uganda, knowledge on HIV/AIDS is necessary but not sufficient to address AIDS related stigmas [12]. Other interventions including public health laws; social marketing; anti stigma campaigns; community mobilization; social activism; and use of mass media are needed to change societal beliefs about people living with AIDS [21]. Newer approaches including use of routine VCT integrated in health services, same-day mobile VCT services, and home-based VCT seem to offer promising results. In addition, availability of ART including use of home-based ART may also increase VCT use among men [10-12,15,24].

### Risky sexual behaviour

A significant proportion of the men engaged in risky sexual behaviour including having multiple sexual partners and engaging in unprotected sexual intercourse. Multiple sexual partners were a common practice and appeared to be a manifestation of male dominance in the society. Surprisingly, in spite of the risky sexual behaviour, perception for HIV risk, especially among older men, was very low. In Zambia, individuals willing to seek VCT were more likely to perceive themselves to be at high risk and were more likely to test HIV positive [30]. However, in rural Zimbabwe, motivation for VCT was driven by knowledge and education rather than sexual risk [25]. One possible explanation for the apparent discrepancy between multiple sexual partners and risk perception may be that multiple sexual partners are a societal norm and men do not perceive it as risky sexual behaviour. Secondly, and as discussed earlier, men may lack adequate knowledge on HIV to appreciate that multiple sexual partners increase the risk of HIV infection.

### Willingness for HIV testing and disclosure

The strongest motivation for HIV testing was the desire to know the HIV status and most men were willing to test for HIV together with their wives. However, this willingness may not translate into action because, according to the men, society would consider a man going with his wife to a clinic for an HIV test as rather strange behaviour. The demand for couple testing is still low [31,32]. In Zimbabwe, Machekano [33] showed that only 7% of the men who tested for HIV brought in their wives for HIV testing. Similarly, although most men were willing to disclose their HIV results to their wives, the fear of negative consequences including sexual denial may ultimately pose a barrier [34-36]. Nevertheless, men's strong desire to know their HIV status and their positive attitude to PMTCT and disclosure of HIV results can be used as a springboard for strategies that target men so as to increase their utilization of VCT. Furthermore the VCT programme should make couple testing more attractive, for example, by improving confidentiality and using the home based VCT approach [11].

### Health system factors

The health system factors that influence VCT use among men were identified during the quantitative and qualitative studies. The most important concerns were access to VCT services, confidentiality and quality of HIV results [12]. Access to VCT services and to health services in general is a nationwide problem [37]. The concerns of access overlap with those for confidentiality. Confidentiality was a major issue among the men, to the extent that many would prefer to test for HIV in a distant clinic where the staff did not know them, but which of course would raise issues of access. As has been reported elsewhere, confidentiality is an important factor that may reduce VCT utilization [30,38,39]. Stigma and confidentiality are closely linked and the greater the stigma of a condition, the greater the need for confidentiality. Our findings suggest that there is strong stigma of HIV/AIDS in Bukonzo West prompting the men to seek VCT services that provide greater confidentiality, even if it means seeking services further away and at a higher cost.

Men also have concerns about the quality of HIV results. This concern is not about the performance of the HIV test but rather about the integrity of the health workers in providing accurate HIV results. Specifically, men fear that health workers are bribed to falsify HIV results. To complicate the situation even more, men do not believe in discordant HIV results and view this as further evidence of corruption. Another angle to the issue of quality is the high drop out between pre-test counselling and HIV testing. This finding suggests that the group counselling that is routinely offered to the men in clinics may not be effective and there is need for one-to-one HIV counselling.

### Limitations of the study

Household size could theoretically have biased the sampling strategy towards men in households with fewer eligible men. However, this was highly unlikely because of the social cultural practices of the Bakonzo. In most cases, a household contains one adult male because male children who are of age (usually 18 years or more) are not allowed to live under the same roof as their parents and have to build their own homes.

Non-response was estimated to be 14% and may have introduced bias in the measurement of the prevalence of VCT and the associated behaviours. Over sampling in all the selected villages (clusters) by randomly selecting 25 respondents instead of the 21 (initially stated) was done in order to increase the sample size.

The study was community-based and heavily relied on the self-report by the respondents. This may have led to reporting bias because of the reluctance to disclose sensitive behaviours such as sexual activity. The lack of an association between sexual behaviour and VCT use in our study may reflect the problem of measuring sexual behaviour. It is also possible that some respondents reported HIV testing behaviours, which they thought were socially desirable but that were not necessarily factual. In addition, recall bias may have occurred particularly with frequently occurring activities.

## Conclusion

Our study showed that VCT use among men in Bukonzo West, Kasese district was low. The major barriers to VCT use among men were poor utilization of VCT services due to poor access, stigma and confidentiality of services. In order to increase VCT use among men, the VCT programme needs to address HIV stigma and improve access and confidentiality of VCT services. Among the more promising interventions are the use of routine counselling and testing for HIV of patients seeking health care in health units, home based VCT programmes, and mainstreaming of HIV counselling and testing services in community development programmes.

## Competing interests

The authors declare that they have no competing interests.

## Authors' contributions

FMB participated in the conception, design, and implementation of the study, statistical analysis, interpretation and drafting of manuscript. SNS, SB, JNK and CASK participated in study conception, design, interpretation and drafting of the manuscript. All authors read, edited and approved the final manuscript.

## Pre-publication history

The pre-publication history for this paper can be accessed here:


